# Discovery of a Novel Molecule that Regulates Tumor Growth and Metastasis

**DOI:** 10.1100/tsw.2008.152

**Published:** 2008-12-14

**Authors:** Chery A. Whipple, Arthur D. Lander, Murray Korc

**Affiliations:** ^1^ Department of Medicine, Department of Pharmacology and Toxicology, and the Norris Cotton Comprehensive Cancer Center, Dartmouth Hitchcock Medical Center and Dartmouth Medical School, Hanover, NH, USA; ^2^ Department of Development and Cell Biology, University of California, Irvine, USA

**Keywords:** Glypican-1, GPC1, pancreatic cancer, PDAC, angiogenesis, metastasis, tumor growth, microenvironment

## Abstract

The heparan sulfate proteoglycan, Glypican-1 (GPC1), significantly impacts the growth of pancreatic cancer cells *in vivo* and markedly attenuates tumor angiogenesis and metastasis in athymic mice. Interestingly, both cancer cell–derived and host-derived GPC1 play an important role in tumor development and spread. These data suggest that GPC1 may be a valid therapeutic target for pancreatic cancer.

Pancreatic ductal adenocarcinoma (PDAC) is a highly metastatic and biologically aggressive malignancy that is the fourth leading cause of cancer death in the U.S. with a 5-year survival rate of less than 5%. Since PDAC is usually diagnosed at an advanced stage, after the tumor has already metastasized and is no longer operable, the number of new PDAC cases diagnosed each year is nearly equivalent to the mortality rate[1]. Late detection is a result of a lack of specific symptoms, difficulty in visualization of early lesions, lack of specific and sensitive diagnostic serum markers, and the highly metastatic nature of this disease[2]. In addition, PDAC displays a wide range of genetic and epigenetic alterations, including the aberrant expression of several key regulators of angiogenesis (blood vessel growth) and invasion, a resistance to chemotherapy and radiotherapy, and a high propensity to metastasize even when small and undetectable[2,3,4].

In a recent study published in the *Journal of Clinical Investigation*, it was shown that a common growth factor coreceptor, Glypican-1 (GPC1), is abnormally abundant in pancreatic cancer and that the loss of GPC1 results in reduced tumor growth, angiogenesis, and metastasis[5]. This work builds on previous studies that have shown that GPC1 enhances and in some cases is required for the efficient binding of several heparin-binding growth factors (HBGFs), including fibroblast growth factor (FGF2), vascular endothelial growth factor (VEGF), placental growth factor (PlGF), and transforming growth factor-β (TGF-β) to their respective receptors, thereby modulating their mitogenic and angiogenic effects on cancer and endothelial cells (Fig. 1)[6,7,8,9,10].

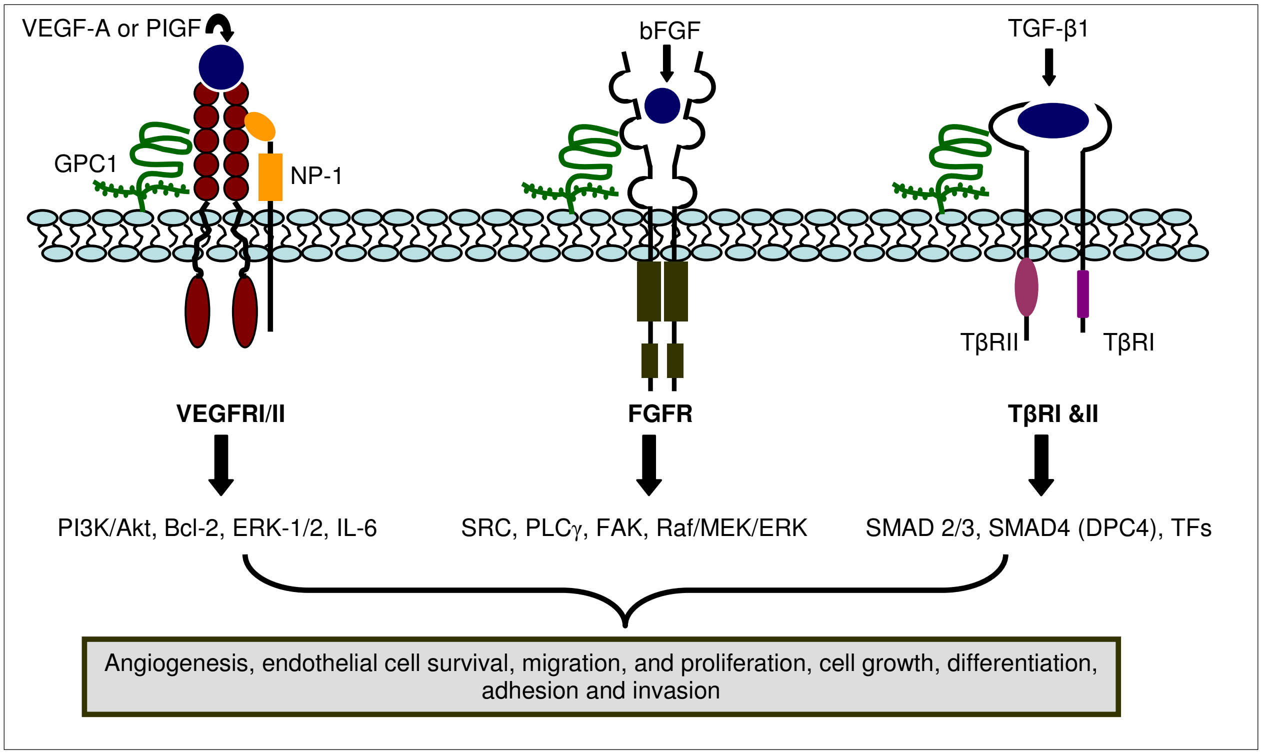

**FIGURE 1.** Example GPC1 signaling pathways. GPC1 acts as a coreceptor in several important growth signaling pathways, including VEGF, FGF, and TBF-β, all of which have a dramatic impact on angiogenesis and cell adhesion, migration, and differentiation.

GPC1 is covalently anchored to the plasma membrane of epithelial cells, fibroblasts, and endothelial cells, and contains a highly conserved globular domain as well as several heparan sulfate sugar side chains that aid in the protein interactions of GPC1 (Fig. 2). Although it is unknown if structural differences exist between the GPC1 present on normal cells and that found on tumor cells, it has been shown that not only is GPC1 dramatically overexpressed in PDAC, but it is also shed into the tumor microenvironment by the cancer cells[5,8,9,10]. It is believed that this shed GPC1 enhances the storage of growth factors and allows for increased growth factor signaling. Collectively, these data suggest that GPC1 may play a pivotal role in cancer progression by influencing tumor growth and metastasis through its signaling within the tumor microenvironment and between cancer cells.

In order to more clearly delineate the importance of GPC1 in pancreatic tumor growth, the researchers reduced the level of GPC1 in cancer cells grown in cell culture. Interestingly, it was found that the cells displayed reduced growth. Next, when these cells were transplanted into immunocompromised mice (mice that lack a thymus gland so the cancer cells will not be rejected), the tumors that developed were not only significantly smaller, but also grew more slowly and had reduced angiogenesis and metastasis when compared to control mice. In a separate experiment, the researchers wanted to determine the role of GPC1 in the host environment. This is of particular interest because GPC1 is found in many tissues, and the microenvironment surrounding tumors has been shown to support tumor growth and metastasis. Thus, in order to study the role of host GPC1, the GPC1 gene was deleted in mice, and these GPC1–/– mice were then crossed with athymic mice. After breeding for 2 years to create a homogeneous population of athymic mice that either expressed or was deficient for GPC1, human cancer cells were introduced into the pancreas. These host mice (devoid of GPC1) developed dramatically smaller pancreatic tumors that were less angiogenic and less metastatic than mice with normal levels of GPC1, providing supporting evidence for the importance of both cancer cell–derived and host-derived GPC1 in stimulating tumor growth and metastasis.

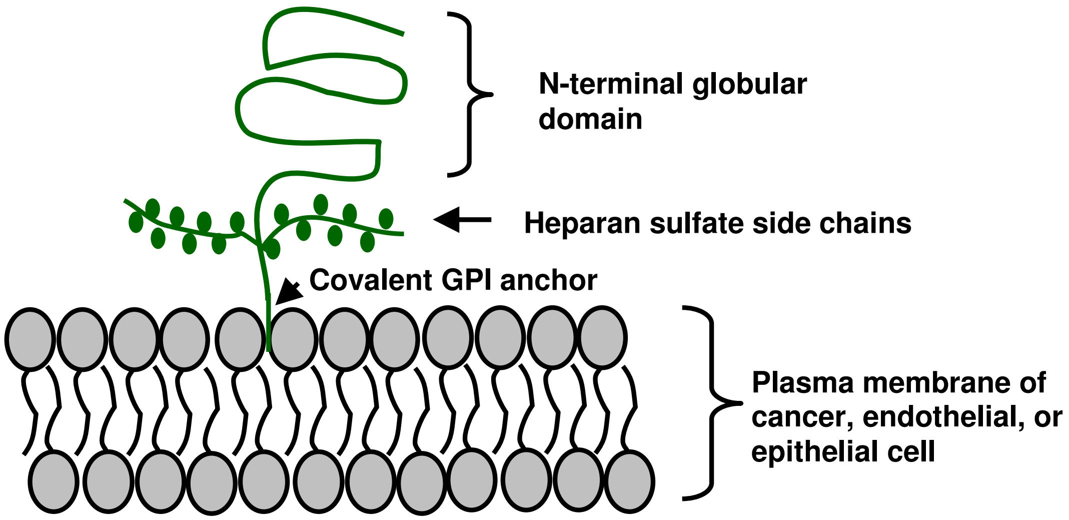

**FIGURE 2.** Schematic diagram of the GPC1 protein structure. Major features include the N-terminal globular domain, which contains several conserved cysteine residues that form intramolecular disulfide bonds, the C-terminal heparan sulfate side chains important for the interaction with signaling molecules, and the glycosyl-phosphatidylinositol (GPI) anchor, which covalently links GPC1 with the plasma membrane.

Given that PDAC is a highly aggressive cancer that displays aberrant angiogenesis and is often metastatic, a better understanding of the mechanisms that contribute to tumor development and growth, and to cancer cell spread, will aid in the elucidation of more effective treatment options for this aggressive malignancy. Since GPC1 plays a pivotal role in tumor angiogenesis and growth, it is plausible that the targeting of GPC1 could significantly impact tumor development and growth. In addition, antiangiogenic therapy, in combination with targeting GPC1, may help to normalize the tumor blood vessels and enhance the efficacy of current chemotherapy and radiation therapies. While there is still more to be discovered about the role of GPC1 in the development and progression of PDAC, especially in regards to the exact pathways it influences to regulate tumor growth and metastasis, the present findings suggest that targeting cancer cell and/or host GPC1 may offer a novel therapeutic approach in PDAC.
